# The Use of Capacitive and Resistive Energy Transfer in Postpartum Pain Management in Women after Perineal Trauma

**DOI:** 10.3390/jcm12186077

**Published:** 2023-09-20

**Authors:** Joanna Siereńska, Zofia Sotomska, Dorota Madej-Łukasiak, Piotr Wąż, Magdalena Emilia Grzybowska

**Affiliations:** 1 Department of Gynecology, Obstetrics and Neonatology, Medical University of Gdańsk, Smoluchowskiego 17, 80-214 Gdańsk, Poland; mgrzybowska@gumed.edu.pl; 2 Independent Team of Physiotherapists, University Clinical Center, Al. Zwycięstwa 30, 80-219 Gdańsk, Poland; zofia.barcikowska@gumed.edu.pl; 3 Department of Obstetrics and Gynecology, Gynecological Oncology and Gynecological Endocrinology, University Clinical Center, Smoluchowskiego 17, 80-214 Gdańsk, Poland; dmadejlukasiak@gmail.com; 4 Department of Nuclear Medicine, Medical University of Gdańsk, Smoluchowskiego 17, 80-214 Gdańsk, Poland; phwaz@gumed.edu.pl

**Keywords:** vaginal delivery, perineal injuries, postpartum, radiofrequency, pain, physiotherapy

## Abstract

Perineal pain occurs in 97% of women with episiotomy or first- and second-degree perineal tears on the first day after delivery. The study aimed to assess the impact of capacitive and resistive energy transfer (TECAR) on perineal pain and discomfort in the first two postpartum days. The prospective randomized double-blind study was performed with the pain and discomfort assessment using the Visual Analogue Scale at baseline and after both TECAR interventions. Characteristics data, delivery information, and the number of painkillers taken were collected. The assumed significance level was α < 0.05. The study included 121 women with a mean age of 30.7 ± 4.2 years and a median BMI of 26.1 kg/m^2^ (24.1; 28.9). Pain reduction at rest, when walking, and discomfort reduction when walking were significantly higher in the TECAR group compared to the sham group (*p* < 0.05). After the first TECAR intervention, significant reduction in all measured parameters was observed in the study group (*p* < 0.03), whereas in the control group, it was observed in pain and discomfort while sitting (*p* < 0.04). The amount of ibuprofen taken on the second day was significantly reduced in the study group compared to the first day (*p* = 0.004). TECAR has been shown to provide more immediate and significant reduction in perineal pain and discomfort.

## 1. Introduction

Perineal injuries are quite common, especially during the first delivery. First- and second-degree perineal tears occur in up to 22.8% of women giving birth [1], while third- and fourth-degree tears occur in 1.8–5.9% of cases [2,3]. Perineal injuries can result in pain and increased perinatal bleeding, and contribute to an increased risk of postpartum depression, the onset of postpartum dyspareunia, and reduced quality of sexual function in women [4,5]. During the first day after a vaginal delivery, perineal pain occurs in up to 97% of women who have had an episiotomy, 95% of women with first- or second-degree perineal tears, and 75% of women with an intact perineum. Approximately 40% of women with an intact perineum still have pain at the end of the first week of postpartum, compared to 60% of women with a first- and second-degree tears, 70% of women with an episiotomy, and up to 90% of women who experienced a third- or fourth-degree tear [6].

Any tissue damage causes the release of peptides, lipids, and inflammatory mediators. These include histamine, bradykinins, prostaglandins, serotonin, and substance P, which stimulate the activation of primary nociceptors that transmit information via A and C nerve fibers to the dorsal horn of the spinal cord in the central nervous system [7]. From the level of the spinal cord, the nociceptive impulse can be inhibited, for example, by descending neurotransmitters modulating serotonin and endogenous opioids [8]. Pain is complex—it is a cognitive, physical, and emotional experience all at once. It can be affected by environmental, cultural, and religious/spiritual conditions. The neuromatrix theory of pain assumes that pain perception is a mind, body, and spirit phenomenon and that past experiences influence our perception of ourselves at any given time [9,10]. A woman’s perception of herself during childbirth, sense of control over pain, and a conscious approach to delivery may affect her perception of pain in the postpartum period [11]. The postpartum period is therefore an opportunity to analyze a woman’s childbirth experience. Entering into a dialogue with women during the postpartum period regarding pain and their needs related to it can help establish pain management strategies [12].

The methods of managing postpartum pain are quite limited due to breastfeeding and are currently mainly based on the use of paracetamol and non-steroidal anti-inflammatory drugs (NSAIDs), such as ibuprofen or ketoprofen [13,14]. It has been shown that pharmacological methods do not always provide complete pain relief, so it would be worth considering supplementing postpartum pain management with non-pharmacological methods [15]. According to research, non-pharmacological methods, such as the use of cold compresses or Transcutaneous Electric Nerve Stimulation (TENS), effectively relieve perineal pain after delivery, especially if it results from edema [6,15]. Another method that could be used for pain relief is Capacitive and Resistive Energy Transfer (TECAR). Although this method has not yet been validated in the early postpartum period, based on other studies, the mechanism of action of high-frequency currents and knowledge of the physiological processes of tissue healing suggest that this method may also be effective for postpartum perineal pain [16]. It has been reported that it can significantly reduce the amount of pain medications taken and reduce the discomfort when walking during the first days after delivery [16].

TECAR is a method that uses a high-frequency alternating current that is administered by using an applicator applied to the skin; otherwise referred to as radiofrequency (RF), thus far it has been most often used in sports, especially after sports injuries for rapid pain relief and smoother regeneration or in the management of lower back pain [17,18], as well as in urology [19]. The effectiveness of the use of radiofrequency in Peyronie’s disease (fibrous inelastic scar of the penis) in men has been proven. The researchers achieved a significant reduction in pain as well as a reduction in erectile dysfunction in the subjects [19]. The diathermy, from which TECAR is derived, was first reported in 1949 and was concerned with the use of thermal application to the perineum after trauma and perineal suturing to accelerate healing and for wound infection after cesarean section [20]. TECAR can be used in two modes: capacitive and/or resistive. The capacitive mode (CET) has a superficial effect by focusing energy on soft tissues, such as muscles, vessels, and the lymphatic system. On the other hand, the resistive mode (RET) penetrates deeper tissues, reaching the fibrous structures. TECAR relies on the oscillation of energy at specific frequencies, generating therapeutic heat in the tissues [21]. Initial reports show that this method has various effects at the cellular level [22,23,24,25]. It may affect the change and mobilization of fats in the early stages of adipogenesis [24]. The high-frequency current improves the regeneration of connective tissue by increasing the number of mesenchymal cells without reducing the ability of stem cells to differentiate. Therefore, TECAR may be a complementary therapy to healing processes [23]. The impact of electrotherapy has also been broadly described for treating lesions, such as pressure ulcers. Although the effects are not fully understood, evidence shows that it improves blood flow, oxygenates cells, reduces edema, leads to pain relief, and positively affects growth factors in the skin [20,25,26,27]. The high-frequency current generates heat in the tissues, stimulating fibroblasts and growth factors that lead to the remodelling of collagen and elastin as well as the accumulation of hyaluronic acid [26].

The study aimed to assess the impact of capacitive and resistive energy transfer on perineal pain levels, perineal discomfort, perineal edema, and the number of analgesics ingested in the first two days after delivery in women who had an episiotomy or perineal tears during the delivery.

## 2. Materials and Methods

The study was conducted at the University Clinical Center in the Clinic of Obstetrics and Gynecology, Gynecologic Oncology, and Gynecologic Endocrinology. Participation in the study was offered to women who had a vaginal delivery with a perineal injury (episiotomy or perineal tears). The study received the consent of the Independent Bioethical Committee for Scientific Research at the Medical University of Gdańsk, no. NKBBN/380/2022. The respondents obtained information on the confidentiality of data collected by researchers and the purpose of their use. The research was conducted over a period of three months between August and November 2022.

### 2.1. Eligibility Criteria

Inclusion criteria included vaginal delivery (including delivery with the use of vacuum or forceps), perineal injury (episiotomy and/or perineal tear) with present perineal pain and discomfort, the ability to sign informed consent to participate in the study, and no contraindications to TECAR therapy. Exclusion criteria included delivery by cesarean section, delivery with complete perineal protection, absence of perineal pain and/or discomfort, inability to sign an informed consent for participation in the study, and contraindications to TECAR therapy. Contraindications to high-frequency current therapy include the presence of electronic devices in the body, coagulation disorders (hemophilia, von Willebrand disease, use of anticoagulants), burns in the examined area, malignancy, severe hypertension or hypotension, and thrombophlebitis.

### 2.2. Examination, Data Collecting

Clinical and personal data (age, body mass index, parity) and information regarding the delivery (length of the first and second stages of labor, single or multiple pregnancies, use of vacuum or forceps, type of perineal trauma, the weight and length of the newborn, the position of the infant at birth, method of perineal suturing) were collected from subjects meeting the inclusion criteria. Additionally, women were asked about pain during intercourse and the presence of urinary incontinence, as well as its severity before childbirth, using an Incontinence Severity Index (ISI) [28]. The number of painkillers (paracetamol, ibuprofen, or others) taken by subjects during the first two days after delivery was recorded. A transvaginal examination was performed by a physiotherapist to assess the strength of the pelvic floor muscles (Modified Oxford Scale from 0 to 5), as well as their endurance (up to 10 s) and reaction timing (up to 10 repetitions) according to the abbreviated PERFECT scheme (Power, Endurance, Fast Contractions) [29].

### 2.3. Intervention

Using the Random Number Generator application, the subjects were randomized into two groups: the study and the control (sham). A double-blind method was used. Subjects with a generated even number were assigned to the study group and those with an odd number were assigned to the sham group. The device did not have a function to randomize the procedure. Women enrolled in the study did not know which group they were randomized into until the end of the study. To blind the trial, the device was paused and covered from view for the tested participant.

The athermal current intensity was set before the start of the examination so that the subjects remained blinded to their group assignment. Due to the use of lower current intensity to achieve the athermal effect, the treatment time was extended to obtain the same effect as with a shorter time and higher intensity, concerning the previous studies on the pelvic floor and sports physiotherapy and data from the TECAR training course [16,30]. The Winback Back S1 device was used in the study. The maximum power of the device for both modes was 100 W. In the study, 20% of CET mode power and 10% of RET mode power were used due to the sensation of the subjects. The frequency of the device’s currents was 500 kHz and 300 kHz. The company, MedeInmed, provided the equipment for the duration of the study. The company, MedenInmed, had no role in study design, data collection, and analysis, nor the decision to publish or the preparation of the manuscript.

**Figure 1:**
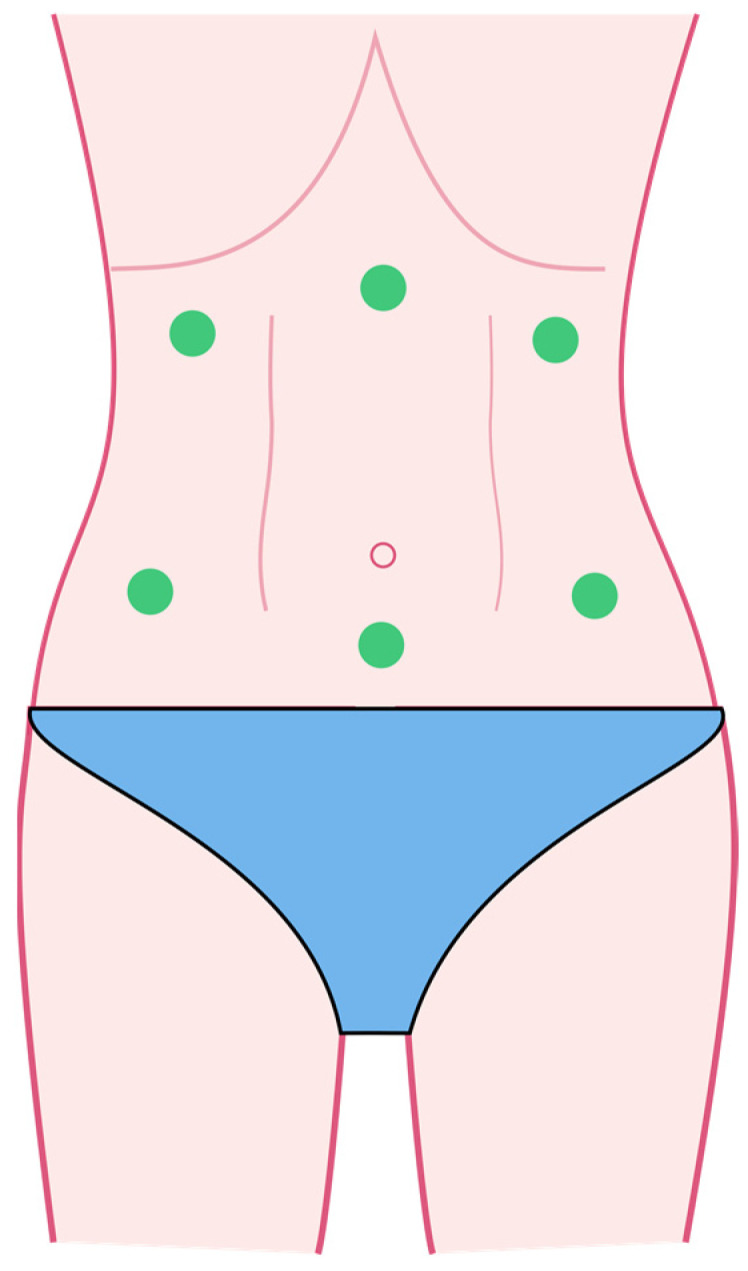
Abdominal phase of the TECAR procedure.

**Figure 2:**
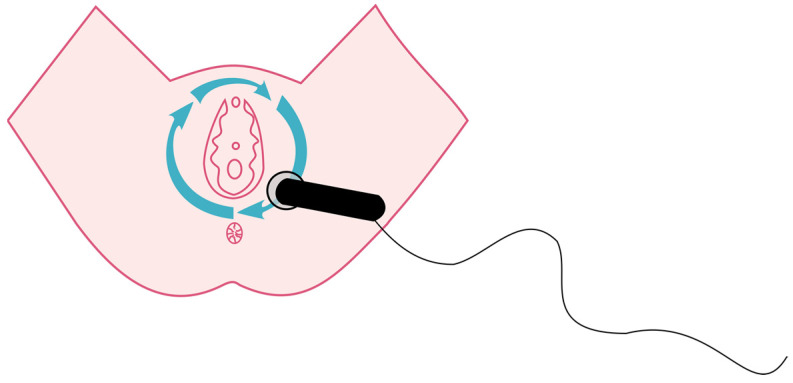
Perineal phase of TECAR procedure—RET.

**Figure 3:**
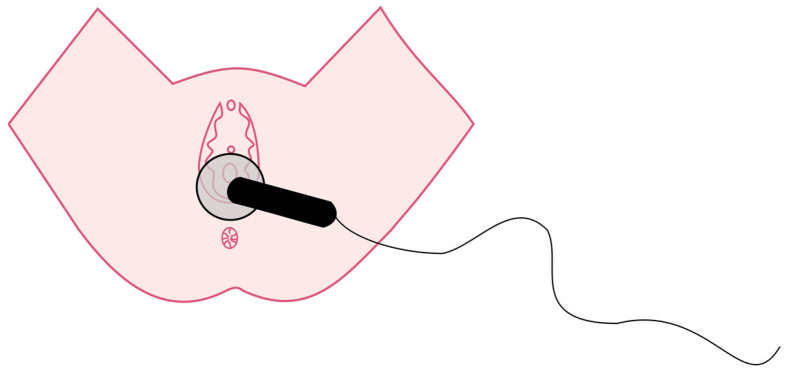
Perineal phase of TECAR procedure—CET.

**Figure 4:**
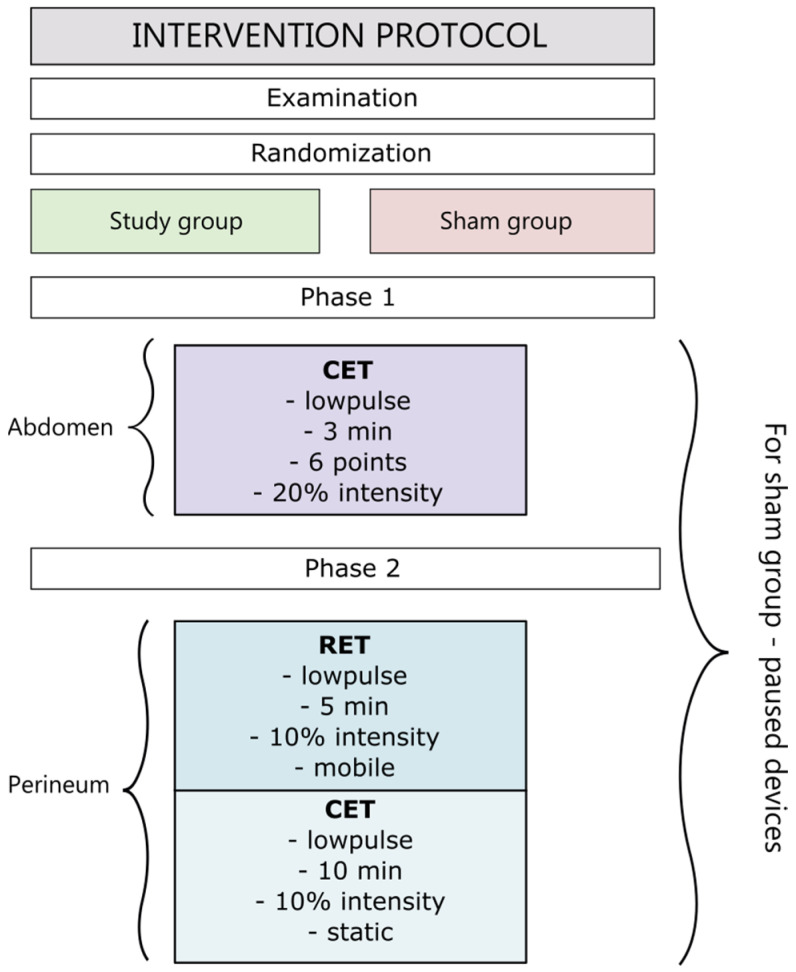
Intervention protocol flow chart.

The study group received an intervention consisting of two phases on the first and second postpartum days. In the first phase, the CET low pulse mode was used for drainage purposes on the abdominal integuments to stimulate the chyle cistern. The phase lasted 3 min and the applicator was applied three times at 6 points for 10 s (Figure 1). In the second phase, using an applicator on the perineum, two types of pulses were applied: RET and CET, both in the low-pulse mode. The RET mode was used for 5 min with circular movements (mobile function) in the perineal area (Figure 2). The CET mode was applied for 10 min with an immobilized applicator (static application) (Figure 3). The sham group received the same intervention as the study group using a device pause. The flowchart of the study is shown in Figure 4.

On two consecutive days, study participants rated their perineal pain and discomfort on a Visual Analogue Scale (VAS) rating from 0 to 10. The following parameters were assessed: pain at rest, pain while sitting, and pain during walking, as well as discomfort during sitting and walking. The first assessment of pain and discomfort, and thus the time of enrollment, occurred within 36 h after delivery (patients who gave birth 1 day back were considered for inclusion). TECAR procedures were performed in the morning and early afternoon—before lunch (consumption of a large meal)—so that bowel fullness would not affect the abdominal part of the procedure. A second and third assessment of pain and discomfort was performed on two consecutive mornings. The study was double-blinded. The external evaluator of the effects of therapy was blinded to the treatment group. The principal investigator asked questions about pain and discomfort before randomization, i.e., before assignment to a specific group. It was not possible to blind the principal investigator herself, given the technical capabilities and the need to involve the physical therapist in performing the procedure on the subjects.

### 2.4. Statistical Methods

Basic statistics, i.e., mean, median, standard deviation, and quartile values, were calculated for quantitative variables. The Shapiro–Wilk test determined whether the values of the analyzed variables came from a population with a normal distribution. The differences between the groups of quantitative variables were tested using the student’s *t*-test or Wilcoxon rank-sum test. The Kruskal–Wallis and Friedman tests were used to compare more than two data groups. For qualitative variables, the basic statistics were the frequency of the analyzed categories. The basic test comparing the relationship between the studied qualitative variables was the χ^2^ test and the Fisher test for independence. The distributions of quantitative variables in graphical form are represented by boxplots; they show distribution parameters, such as the first and third quartiles, the median, and the interquartile range values. In addition, the presented boxplots contain information on the *p*-values for the Wilcoxon tests (in the case of comparing two distribution values). If more than two distributions of values were compared, the figures show *p*-values for selected post-hoc tests related to the Friedman or Kruskal–Wallis tests. The assumed significance level was α < 0.05.

## 3. Results

### 3.1. Characteristics of the Study Groups

In total, 121 women were enrolled, of whom 59 were randomized to the sham group and 62 to the study group. The mean age of the women was 30.7 ± 4.2 years and the mean BMI was 26.1 kg/m^2^. Among the examined women, 60 were primiparas and 61 were multiparous. In the study group, the median duration of the first stage of labor was 7 (4.1; 10) hours, and the second stage of labor was 1 (0.8; 1.5) hour, while in the sham group, it was 7 (4; 11.8) and 1 (0.5; 1.5) hours for phase I and II of labor, respectively. The characteristics of the groups are presented in Table 1.

### 3.2. Perineal Injuries

All subjects had a vaginal delivery and perineal trauma (episiotomy and/or tears in grades I–III), as presented in Table 2. All study participants gave birth to children in the longitudinal, cephalic position. A total of 105 (86.8%) women underwent episiotomy, 21 (17.4%) had first-degree perineal tears, 8 (6.6%) had second-degree tears, and 4 (3.3%) had third-degree perineal tears. The study and sham groups did not differ in terms of the type of perineal injury. Most of the women in the study (86.8%) had episiotomy. Women with grade I–II perineal tears were a minority in the groups due to the frequent absence of pain/discomfort in this case.

### 3.3. Medications Intake

In the study group, on the first day, women took a median of 0.4 (0; 0.8) g of ibuprofen and 0 (0; 1) g of paracetamol. On the second day, they took 0.4 (0; 0.4) g of ibuprofen and 0 (0; 1) g of paracetamol. In the sham group, the mean analgesics intake was as follows: for ibuprofen 0.4 (0; 0.4) and 0.4 (0; 0.4) g, for paracetamol 0.2 (0; 1) and 0 (0; 1) g. In the within-group analysis, the amount of ibuprofen taken on day two compared to day one in the study (intervention) group was significantly reduced (*p* = 0.004) (Table 3).

### 3.4. Pain and Discomfort Level Outcomes

Before TECAR application, the median pain at rest was 5 (3; 6) in the study group and 3 (2; 5) in the sham group, which was assessed with VAS. Median pain while sitting was rated as 6 (4; 7) and 5 (3; 6), and during walking it was 3.5 (2; 5) and 3 (0; 4.5) in the study and sham groups, respectively. In the study group, the baseline discomfort while sitting was 6 (4.2; 7), and while walking was 4.5 (3; 6), compared to the sham group, which was 5 (4; 6), and 3 (2; 5). These differences between the study and sham groups were statistically significantly different (Table 4). On subsequent days—after the first and second TECAR treatment—there were no statistical differences between the study and sham groups.

#### 3.4.1. Between the Study Groups Analysis

Due to the significant differences in VAS levels at baseline across parameters between the groups, the change in VAS was evaluated for further analysis. Statistically significant differences were found between the groups in pain and discomfort reduction during the analysis (Table 5). The reduction between the second TECAR intervention and the baseline for pain at rest was significantly higher in the study group (*p* = 0.045), as well as the reduction between the first intervention and the baseline for pain at rest (*p* = 0.049) and pain while walking (*p* < 0.01). The reduction of the discomfort during walking between the second and the first TECAR interventions and the baseline was also significantly higher in the study group (*p* = 0.049 and *p* = 0.02) (Table 5).

#### 3.4.2. Within the Study Groups Analysis

**Figure 5:**
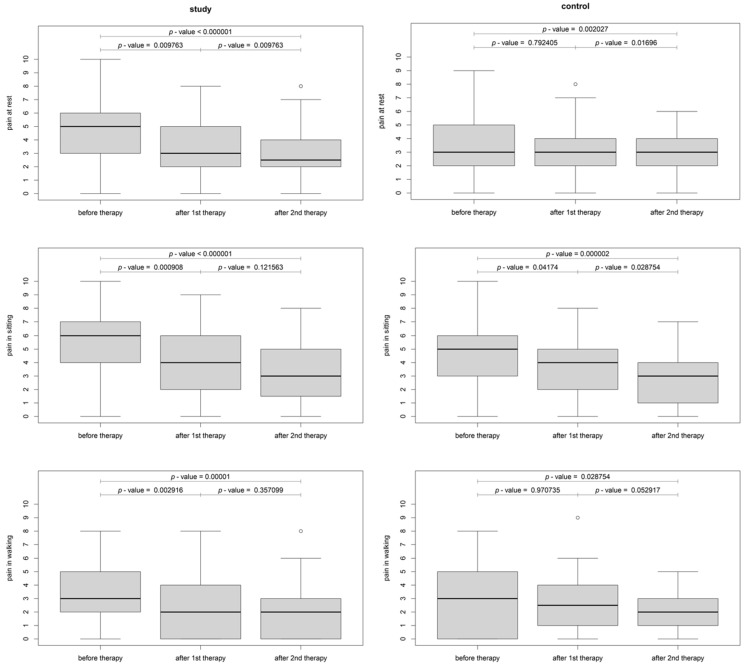
Boxplot of pain at baseline, first and second TECAR interventions in the study and sham groups ^w^ (^w^—Wilcoxon rank-sum test with continuity correction).

**Figure 6:**
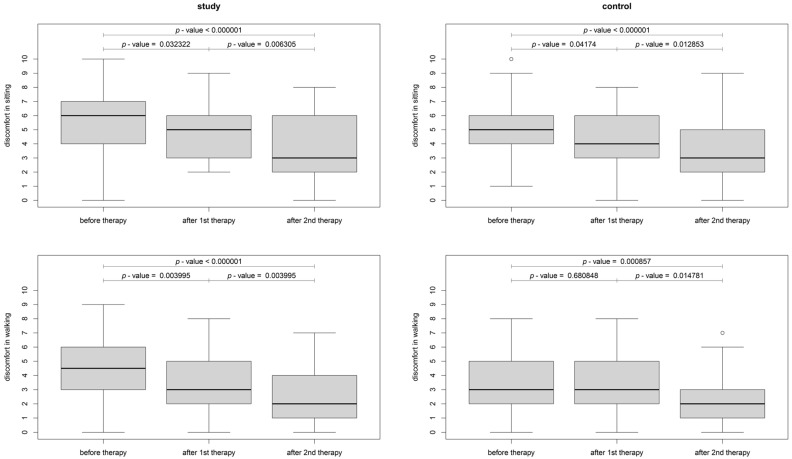
Boxplot of discomfort at baseline, first and second TECAR interventions in the study and sham groups ^w^ (^w^—Wilcoxon rank-sum test with continuity correction).

The change in pain and discomfort within the study and sham groups was also analyzed. In the study group, a significant difference between baseline and after the first treatment was detected for pain at rest (*p* < 0.01), pain while sitting (*p* < 0.001), and pain during walking (*p* < 0.003). Therefore, there was an improvement observed in all pain levels after the first (*p* < 0.01) and after the second (*p* < 0.0001) intervention when compared to the baseline (Figure 5). In the sham group, there were significant differences in pain between baseline and after the first treatment while sitting (*p* = 0.04), while pain at rest and pain during walking differed significantly between the baseline and second treatment (the improvement occurred only after the second day) (Figure 5). As for the discomfort assessment, in the study group, both discomfort while sitting and walking differed significantly between baseline and the first and second treatments (*p* < 0.04). In the sham group, improvement occurred after the first intervention for discomfort while sitting and after the second intervention for discomfort when walking (Figure 6). Between the first and second TECAR treatments, there was no significant change in pain parameters in the study group except for pain at rest (*p* = 0.01), while there was a significant reduction of discomfort in both discomfort when sitting (*p* = 0.006) and walking (*p* = 0.004). For the control group, there was a significant reduction in pain at rest (*p* = 0.02) and when sitting (*p* = 0.03), as well as discomfort when sitting (*p* = 0.01) and walking (*p* = 0.02) (Figure 5 and Figure 6).

## 4. Discussion

This study showed that the use of high-frequency current therapy can have a positive effect on reducing perineal pain and discomfort in the first days after vaginal delivery with perineal trauma. A previous study conducted by Bretelle et al. [16] has not found this method beneficial for relieving postpartum perineal pain at rest, when sitting, and when walking [16]. However, in their study, only women with a pain VAS of >4 were enrolled [16], whereas in our case, a pain VAS of >1 was assumed. To date, positive effects of TECAR therapy on pain relief after sports injuries or in Peyronie’s disease have been observed [17,18]. So far, one review has been published on the use of TECAR in pelvic structural dysfunction [30]. The review identified two studies on pain assessment as promising; Fernández-Cuadros et al. were successful with participants with chronic pelvic pain and dyspareunia. They observed a significant reduction in pain and improvement in pelvic floor muscle strength. Another study investigated the effect of diathermy on painful menstruation in endometriosis. Five participants were treated for three menstrual cycles, with improvements in the intensity of painful menstruation, sexual pain, and myofascial pain. These results cannot be considered generalizable due to the small sample size and the lack of a control group, and encourage the design of a larger study [30]. 

Although the use of the VAS for pain assessment can be controversial due to the subjective nature of the scale, its use has been confirmed as a reliable indicator of the treatment effect on labor pain [31]. For a better assessment, it is recommended to accompany the VAS with a verbal description to assess the therapeutic effect more accurately [31]. In fact, the VAS scale, which allows a proper comparison, has been used in most of the previous studies on the use of TECAR [16,30].

In our study, two interventions were performed on a daily basis due to the time limitation of the inpatient hospital stay of study subjects (the standard hospital stay after vaginal delivery is two days) [32]. Other studies have used 1–3 TECAR sessions for a group of healthy individuals and 6–24 sessions (2–6 times per week) for dysfunctions such as lower limb lymphoedema or Achilles tendonitis [33]. As a consequence of this difference, the results may not have been as spectacular as in other studies [33]. Other studies on the use of TECAR in sports reported that the intervention lasted between 2 and 8 weeks with intervals of a few days to a week [18]. In the case of Peyronie’s disease, the intervention lasted 3 days, with 3 treatments each day [19]. Therefore, when analyzing TECAR results and comparing different studies, it is important to evaluate the treatment regimen in detail.

In the present study, TECAR did not reduce the amount of analgesic taken in the first two days after delivery, which differs from the results of the previous study [16]. In the previously reported study, the mean cumulative dose of paracetamol, in the first two days after delivery in the TECAR group, was 0.98 ± 0.17 g and, in the sham group, it was 1.7 ± 0.27 g (*p* = 0.035), compared with our study, in which the mean paracetamol dose in the groups did not differ significantly and was 1.2 ± 1.25 g and 1.02 ± 1.14 g in the study and sham groups, respectively [16]. Thus, there was no significant change in the use of analgesics in our study compared to the previous study on TECAR in relieving postpartum perineal pain. It should be considered whether the use of pain medication is a good parameter to analyze when evaluating the efficacy of the above method, especially in such a complex process as postpartum. It is worth noting that the use of painkillers is often associated not only with perineal pain but also with pain caused by uterine involution. Therefore, the amount of analgesic intake parameter may not be adequate to control the efficacy of TECAR therapy [34]. In our study, paracetamol and ibuprofen were the most commonly used analgesics during breastfeeding because of safety concerns [35]. Ketoprofen was used in a few cases in our study.

The median pain level of the subjects who experienced perineal trauma was VAS 5 (3; 6) before the intervention: for pain at rest, 6 (4; 7); for pain while sitting; and 3.5 (2; 5) for pain while walking. In the previous studies, slightly different parameters for episiotomy and second-degree perineal tears were reported, such as 2.75 (2.3) for pain at rest, 4.23 (2.3) when sitting, and 4.28 (2.7) when moving [36,37]. Thereby, the baseline parameters measured in our study were higher (pain at rest and pain while sitting) and slightly lower (pain while walking/moving) than in other cited studies. The median pain level after two interventions was 2.5 (2; 4)—pain at rest, 3 (1.8; 5); pain while sitting, 2 (0; 3); pain while walking for the study group and sham group, 3 (2; 3.8), 3 (1.2; 4), 2 (1; 3) respectively. In the literature, evidence is available regarding the relief of postpartum perineal pain using other methods, such as cold compresses on the perineum, hydrotherapy, or painkillers [6,38,39]. The demonstrated changes in pain parameters before and after TECAR therapy were similar to the effects obtained with cold compress application, and, as is known, cold compresses are a well-studied and commonly used method of relieving perineal pain. Before the application of the compresses, the group had a mean pain of 6.73 (SD 1.68), while after two applications, a mean pain of 2.01 (SD 1.02) was observed. However, the pain decreased for a limited time after the intervention [6]. On the other hand, using warm hydrotherapy on the first postpartum day also resulted in a reduction in pain, but it was lower than in the present study [37]. In a review of the literature on the effect of NSAIDs on postpartum perineal pain, a decrease in pain was also obtained—up to 50% [38]. Therefore, it can be concluded that TECAR can be considered another effective method in relieving postpartum perineal pain apart from cold compresses and pain medications.

In some women, postpartum pain is associated with the onset of postpartum depression. The literature reports that labor pain and acute postpartum pain may be associated with acute and persistent symptoms of postpartum depression [39]. Effective postpartum pain relief appears to be a critical component of a woman’s postpartum care. With appropriate pain management, there is an opportunity to increase a woman’s focus on motherhood’s responsibilities and reduce the risk of chronic pain and symptoms of postpartum depression [36,39]. However, data are still lacking regarding non-pharmacological, complementary therapies, such as physical therapy or psychological support [35].

## 5. Conclusions

To our knowledge, this is the second study on the effect of TECAR on postpartum perineal pain in the first days after vaginal delivery. Based on the above results, TECAR has been proven to have a more immediate reduction in perineal pain and discomfort. After its application, the reduction in perineal pain and discomfort, especially during rest and walking, was significant. However, it did not reduce the subjects’ use of pain medication. The possibility of using supplementary equipment, such as TECAR, which so far has had no detectable side effects, to manage postpartum pain opens the way for even better care and improved quality of perinatal functioning for women.

## 6. Study Limitations

A limitation of our study was the use of reduced current intensity due to the athermal effect to blind the subjects. Another limitation is the duration of hospitalization, which is usually 2–3 days after vaginal delivery and which allowed for a maximum of 2 TECAR applications. Unfortunately, we did not have the conditions to conduct a longer study at our center, and it could be challenging to assemble a group in the post-hospitalization period. A concerning limitation may also be the difference in the baseline measurement of pain and discomfort between the study and the sham groups. The higher pain and discomfort parameters in the study group may have introduced confusion in the proper evaluation of the study results. However, for this reason, the change in pain and discomfort VAS parameters in further analysis was evaluated.

## Figures and Tables

**Table 1:** Participants’ characteristics.

Variables	Study Group (n = 62)	Sham Group (n = 59)	*p*-Value
Age (years)	30.5 ± 3.8	31 ± 4.6	0.58 ^t^
BMI (kg/m^2^)	26 (24.3; 28.4)	26.8 ± 3.8	0.84 ^w^
Primaparas Multiparous	32 (51.6%) 30 (48.4%)	28 (47.5%) 31 (52.5%)	0.78 ^c^
I labor stage length (in hours)	7 (4.1; 10)	7 (4; 11.8)	0.69 ^w^
II labor stage length (in hours)	1 (0.8; 1.5)	1 (0.5; 1.5)	0.20 ^w^
Child weight (g)	3400 (3055; 3582.5)	3478.7 ± 461.9	0.16 ^w^
Child length (cm)	54 (52; 56)	55 ± 2.4	0.08 ^w^
Previous episiotomy	21 (33.9%)	24 (40.7%)	0.56 ^c^
Previous perineal tear	4 (6.5%)	6 (10.2%)	0.85 ^c^

Basic statistics, for quantitative variables—mean ± standard deviation or median (first quartile; third quartile), for qualitative variables—frequency; ^c^—Pearson’s Chi-squared test with Yates’ continuity correction, ^t^—Student’s *t*-Test ^w^—Wilcoxon rank sum test with continuity correction.

**Table 2:** Distribution of perineal injuries among the study participants.

Perineal Injury/Instrumental Delivery	Study Group (n = 62)	Sham Group (n = 59)	*p*-Value
Episiotomy	54 (87.1%)	51 (86.4%)	1 ^c^
1st-degree perineal tear 2nd-degree perineal tear 3rd-degree perineal tear	9 (14.5%) 6 (9.7%) 3 (4.8%)	12 (20.3%) 2 (3.4%) 1 (1.7%)	0.38 ^f^
Vacuum delivery	2 (3.2%)	3 (5.1%)	0.67 ^f^
Forceps delivery	0 (0%)	0 (0%)	1 ^f^

Data presented as number (percentage) n (%); ^c^—Pearson’s Chi-squared test with Yates’ continuity correction, ^f^—Fisher’s exact test for count data.

**Table 3:** The number of analgesics used—within and between group analysis.

Analgesics	Study Group (n = 62)	Sham Group (n = 59)	*p*-Value
**Paracetamol (g)**			
**1st day**	0 (0; 1)	0.2 (0; 1)	0.52 ^w^
**2nd day**	0 (0; 1)	0 (0; 1)	0.53 ^w^
***p-*** **value**	0.33 ^w^	0.11 ^w^	
**Ibuprofen (g)**			
**1st day**	0.4 (0; 0.8)	0.4 (0; 0.4)	0.80 ^w^
**2nd day**	0.4 (0; 0.4)	0.4 (0; 0.4)	0.72 ^w^
***p-*** **value**	**0.004** ^w^	0.08 ^w^	

^w^—Wilcoxon rank-sum test with continuity correction, data presented as median (first quartile; third quartile).

**Table 4:** Comparison of pain and discomfort levels (VAS) between the study groups.

Variables	Study Group (n = 62)	Sham Group (n = 59)	*p*-Value ^w^
**Pain at rest (VAS)**			
Baseline	5 (3; 6)	3 (2; 5)	**0.01**
1st intervention	3 (2; 5)	3 (2; 4)	0.42
2nd intervention	2.5 (2; 4)	3 (2; 3.8)	0.97
**Pain while sitting (VAS)**			
Baseline	6 (4; 7)	5 (3; 6)	**0.02**
1st intervention	4 (2; 6)	4 (2; 5)	0.62
2nd intervention	3 (1.8; 5)	3 (1.2; 4)	0.55
**Pain while walking (VAS)**			
Baseline	3.5 (2; 5)	3 (0; 4.5)	**0.048**
1st intervention	2 (0; 4)	2 (1; 4)	0.61
2nd intervention	2 (0; 3)	2 (1; 3)	0.90
**Discomfort while sitting (VAS)**			
Baseline	6 (4.2; 7)	5 (4; 6)	**0.01**
1st intervention	5 (3.2; 6)	4 (3; 6)	0.16
2nd intervention	3 (2; 6)	3 (2; 5)	0.33
**Discomfort while walking (VAS)**			
Baseline	4.5 (3; 6)	3 (2; 5)	**0.02**
1st intervention	3 (2; 4.8)	3 (2; 4.5)	0.82
2nd intervention	2 (1; 4)	2 (1; 3)	0.49

Data presented as median (first quartile; third quartile); ^w^—Wilcoxon rank-sum test with continuity correction.

**Table 5:** Comparison of pain and discomfort change between the study groups.

Variables	∆ Study Group (n = 62) Me (Min, Max)	∆ Sham Group (n = 59) Me (Min, Max)	*p*-Value ^w^
**Pain at rest**			
2 − baseline *	−2 (−3; −1)	−1 (−2; 0)	**0.045**
1 − baseline **	−1 (−2; 0)	0 (−1.8; 1)	**0.049**
2 − 1 ***	−1 (−1.2; 0)	−1 (−1; 0)	0.86
**Pain while sitting**			
2 − baseline	−2.5 (−4; −1)	−2 (−3; 0)	0.15
1 − baseline	−1 (−3.2; 0)	−1 (−2; 0)	0.13
2 − 1	−1 (−2; 0)	−1 (−2; 0)	0.6
**Pain while walking**			
2 − baseline	−1 (−3; 0)	−1 (−2; 0)	0.08
1 − baseline	−0.5 (−2;0)	0 (−1; 1)	**<0.01**
2 − 1	0 (−1.2; 0)	−0.5 (−2; 0)	0.26
**Discomfort while sitting**			
2 − baseline	−2 (−4; 0)	−1 (−2.8; 0)	0.26
1 − baseline	−1 (−3; 0)	−1 (−2; 0)	0.52
2 − 1	−1 (−2; 0)	−1 (−2; 0)	0.49
**Discomfort while walking**			
2 − baseline	−2 (−3; −1)	−1 (−2; 0)	**0.049**
1 − baseline	−1 (−2; 0)	0 (−1.8; 1)	**0.022**
2 − 1	−1 (−1; 0)	−1 (−2; 0)	0.54

Data presented as median (first quartile; third quartile); *—difference between the second intervention and the baseline; **—difference between the first intervention and the baseline; ***—difference between the second and first intervention, ^w^—Wilcoxon rank-sum test with continuity correction.

## Data Availability

Data supporting reported results will be available on request.
